# Integration of Shallow Gradients of Shh and Netrin-1 Guides Commissural Axons

**DOI:** 10.1371/journal.pbio.1002119

**Published:** 2015-03-31

**Authors:** Tyler F. W. Sloan, Mohammad A. Qasaimeh, David Juncker, Patricia T. Yam, Frédéric Charron

**Affiliations:** 1 Molecular Biology of Neural Development, Institut de Recherches Cliniques de Montréal (IRCM), Montreal, Quebec, Canada; 2 Division of Experimental Medicine, McGill University, Montreal, Quebec, Canada; 3 Program in Neuroengineering, McGill University, Montreal, Quebec, Canada; 4 Division of Engineering, New York University Abu Dhabi, Abu Dhabi, United Arab Emirates; 5 Department of Biomedical Engineering, McGill University, Montreal, Quebec, Canada; 6 McGill University and Genome Quebec Innovation Centre, McGill University, Montreal, Quebec, Canada; 7 Department of Anatomy and Cell Biology, Department of Biology, McGill University, Quebec, Canada; 8 Department of Medicine, University of Montreal, Montreal, Quebec, Canada; The Scripps Research Institute, UNITED STATES

## Abstract

During nervous system development, gradients of Sonic Hedgehog (Shh) and Netrin-1 attract growth cones of commissural axons toward the floor plate of the embryonic spinal cord. Mice defective for either Shh or Netrin-1 signaling have commissural axon guidance defects, suggesting that both Shh and Netrin-1 are required for correct axon guidance. However, how Shh and Netrin-1 collaborate to guide axons is not known. We first quantified the steepness of the Shh gradient in the spinal cord and found that it is mostly very shallow. We then developed an in vitro microfluidic guidance assay to simulate these shallow gradients. We found that axons of dissociated commissural neurons respond to steep but not shallow gradients of Shh or Netrin-1. However, when we presented axons with combined Shh and Netrin-1 gradients, they had heightened sensitivity to the guidance cues, turning in response to shallower gradients that were unable to guide axons when only one cue was present. Furthermore, these shallow gradients polarized growth cone Src-family kinase (SFK) activity only when Shh and Netrin-1 were combined, indicating that SFKs can integrate the two guidance cues. Together, our results indicate that Shh and Netrin-1 synergize to enable growth cones to sense shallow gradients in regions of the spinal cord where the steepness of a single guidance cue is insufficient to guide axons, and we identify a novel type of synergy that occurs when the steepness (and not the concentration) of a guidance cue is limiting.

## Abstract 2

Two gradients are better than one: the axon guidance cues Shh and Netrin synergize to guide growth cones in shallow gradients in which one cue alone would be insufficiently steep to guide axons.

## Author Summary

During development of the nervous system, axons are propelled by the growth cone, a motile structure that is specialized to detect the direction of concentration gradients of guidance cues. Although it is known that commissural axons—those that cross the midline from one side of the nervous system to the other—of the spinal cord are guided by multiple cues simultaneously, it is unclear whether they integrate multiple guidance cues and, if that is the case, the advantage of doing so. In the developing spinal cord, the gradients of the guidance cues are shallow and, thus, their direction is difficult to determine. We hypothesize that under these circumstances, a combination of cues could be used synergistically by the growth cone. To test this hypothesis, we built a microfluidic gradient generator capable of simulating the shallow gradients that growth cones encounter in the developing spinal cord. Using this guidance assay, we demonstrated that commissural axons are best at reorienting themselves in the steepest part of a gradient of Shh or Netrin, two guidance cues that these axons encounter in the spinal cord. We then challenged axons with combined gradients of both cues. At low gradient steepness, we observed synergy in their turning response and in the asymmetry of the shared downstream signaling molecules Src-family kinases (SFKs). We therefore propose a model in which SFKs integrate distinct signaling pathways, and we define this as steepness-limited synergy.

## Introduction

During embryogenesis, axons grow through a complex environment to make specific connections with their targets. The growth cone follows concentration gradients of guidance cues by sensing a difference in receptor occupancy across its width, and it turns to align with its interpretation of the gradient direction. Moreover, multiple guidance cues are often needed to correctly guide axons. For example, commissural axons are initially repelled by bone morphogenic proteins (BMPs) in the dorsal half of the spinal cord [1,2]. They are then attracted by gradients of Netrin-1 [3], Sonic hedgehog (Shh) [4] and vascular endothelial growth factor (VEGF) [5] towards the floor plate. While it isn't understood why multiple guidance cues are needed to guide axons to the same targets, it is clear they are non-redundant, as interfering with each of these pathways individually results in guidance errors [4–8].

Both Netrin-1 [9,10] and Shh [11,12] diffuse from the floor plate cells which secrete them and establish gradients which guide commissural axons [4,10]. Shh signals through its receptor Boc [8], while Netrin signals through its receptor DCC [7,13]. Shh- and Netrin-1-mediated axon guidance also both require Src-family kinase (SFK) activity [14,15], whose asymmetric activation reflects the direction of the external gradient and is sufficient to cause the growth cone to turn [15,16]. While it is known that both Shh and Netrin-1 form gradients, it is not clear how steep the gradients are in vivo and how this steepness influences axon pathfinding in gradients formed by single or multiple guidance cues. Although theoretical chemotaxis modeling has suggested that two overlapping attractive concentration gradients could increase the probability of a cell making a correct decision about the gradient direction [17], this prediction has not been tested experimentally.

There are several potential mechanisms by which multiple guidance cues could collaborate to improve how well the growth cone estimates the direction of the gradient. In one model, the concentration of individual guidance cues is too low to elicit a robust turning response. When the cues are combined, the response is higher than the sum of responses from the same concentration of either cue individually. We will refer to this as concentration-limited synergy, as the concentration of either guidance cue is limiting for the pathway to be engaged. When a second cue is present, there is some crosstalk or convergence between pathways, which overcomes the activation threshold. In an alternative mechanism, which we will refer to as steepness-limited synergy, the concentration of guidance cue present at the growth cone is not limiting; instead, it is the concentration difference of an individual guidance cue across the growth cone that is too small compared to the ambient guidance cue concentration to be accurately detected by the growth cone. When two guidance cues are present, corroborating directional information is supplied and integrated by the growth cone through crosstalk or convergence between the two guidance cue pathways.

We demonstrate that commissural axon guidance errors occur in vivo when the Shh concentration gradient is relatively shallow. We then use a novel microfluidic guidance assay to show the importance of gradient steepness for commissural axon guidance in vitro. We find that a combined gradient of the attractive guidance cues Shh and Netrin-1 can act in steepness-limited synergy to attract axons when the steepness of a single guidance cue is insufficient to guide axons. Mechanistically, we demonstrate that combined Shh and Netrin-1 gradients polarize SFK phosphorylation in the growth cone at the same gradient steepness when the two cues behaved synergistically to attract axons.

## Results

### Shallow Gradients Guide Commissural Axons En Route to the Floor Plate

**Fig 1:**
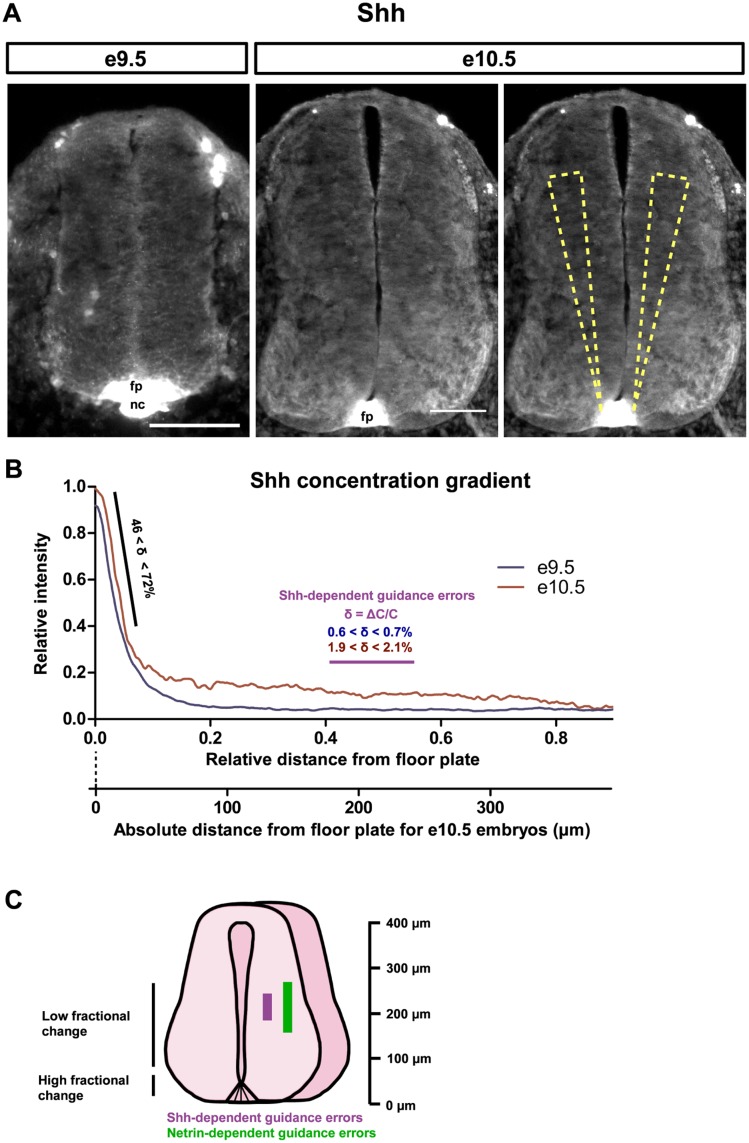
Axon misguidance phenotypes in vivo occur where the Shh gradient is shallow. **(A)** Mouse e9.5 (left) and e10.5 (middle and right) spinal cord cross-sections immunostained for Shh. We measured fluorescence intensity profiles at multiple angles along the cross-section of the spinal cord; angle range indicated by wedges (right). **(B)** Shh concentration was highest at the floor plate and decreased rapidly in the first ~50 μm (relative distance of 0–0.1) from the floor plate. Beyond this steep decline in intensity, there was a residual shallow gradient extending >300 μm, up to a relative distance of 0.8 from the floor plate to the roof plate. 29 sections from two e9.5 embryos and 15 sections from two e10.5 embryos were analyzed. The steepness of the gradient, δ, was defined as the fractional change in concentration (δ = ΔC/C) over a distance of 10 microns, and was 46 < δ < 72% in the steep region close to the floor plate (black line), and 0.6 < δ < 0.7% at e9.5 and 1.9 < δ < 2.1% at e10.5 at a relative distance of 0.41–0.56 from the floor plate, where guidance errors have been reported for mice with mutations in Shh signaling (purple line). The guidance errors that have been reported for mice with mutations in Netrin-1 signaling occupy a similar region of the spinal cord. The absolute distance scale from floor plate is for e10.5. **(C)** Schematic spinal cord showing the approximate relative positions of the regions of high and low fractional change, and the range of guidance errors reported previously. fp, floor plate; nc, notocord. Scale bar (A): 100 μm. See also S1 Fig, S2 Fig, and S1 Table.

To determine the Shh gradient steepness that growth cones of commissural axons are exposed to in vivo, we examined spinal cord cross sections of embryonic day 9.5 (e9.5) and e10.5 mouse embryos, stages when axons are actively being guided towards the floor plate. We visualized the distribution of Shh protein in paraformaldehyde-fixed spinal cords using immunofluorescence with an anti-Shh antibody [18]. The Shh staining present in the floor plate and the spinal cord were not present in *Shh* ^-/-^ embryos (S1A Fig), demonstrating that the antibody specifically recognized Shh. We then measured the fluorescence intensity profiles of the Shh protein gradient along the dorso-ventral axis at several angles for each image (Fig. 1A) and pooled these measurements from multiple embryos to obtain a prototypical gradient profile (Fig. 1B). Shh fluorescence signal was highest at the floor plate and rapidly decreased for approximately 50 μm from the floor plate, followed by a slower decrease for the remainder of the spinal cord. We observed that the gradient profiles were remarkably consistent between embryos (S1B Fig) and that they did not depend on the concentration of the primary antibody (S1C Fig). We then demonstrated that there is a linear relationship between the fluorescence intensity and the concentration of Shh protein (S1D Fig). Furthermore, the gradient profiles were similar whether the measurements were made medially (as in Fig. 1A) or more laterally, overlapping with Tag-1 positive axons (S2 Fig).

Both the concentration (C) and steepness of the gradient can influence axon guidance responses. Because growth cones must be able to determine the direction of a gradient, it is essential that they can sense a difference in concentration across their width. This can be expressed as the absolute change in concentration across a growth cone (ΔC). The fractional change in concentration (δ = ΔC/C) is a measure of the steepness of the gradient across the growth cone, typically estimated at 10 μm [19]. The fractional change is usually expressed as a percentage and reflects the change in concentration across a growth cone relative to the ambient concentration at the growth cone.

Although it is not possible to accurately quantify absolute protein levels in vivo using immunohistological methods, measuring the fractional change in concentration does not require knowledge of the actual concentration of the cues, only the relative concentration of the cue. Thus we estimated the fractional change in concentration using the Shh fluorescence intensity. Within 50 μm of the floor plate (relative distance of 0–0.1 from the floor plate to the roof plate), there is a rapid decrease in Shh, with a fractional change (δ) of 46%–72% (Fig. 1B). In the region beyond 50 μm of the floor plate, the Shh gradient was shallower.

We then determined where along the spinal cord guidance defects occur for commissural axons from mice genetically deficient for Shh or Netrin-1 signaling. We analyzed images from previously reported guidance cue or guidance receptor mutants [4,6–8] and measured the relative distance from the floor plate at which misguided axons begin to deviate from their normal trajectory (S1 Table). For Shh and Netrin-1 signaling dependent defects, guidance errors occurred at a relative distance of 0.35–0.6, which corresponds to 158–270 μm from the floor plate for a spinal cord ~450 μm in height. In the region where Shh dependent errors occur (relative distance of 0.41–0.56), the Shh gradient at e9.5 was very shallow, with a fractional change of 0.6 < δ < 0.7%. At e10.5, when the majority of the commissural axon growth cones are en route from the roof plate to the floor plate, the fractional change in this region was 1.9 < δ < 2.1%, slightly higher than that measured at e9.5 (Fig. 1B). The Netrin-1 gradient has been previously visualized at mouse e10.5 using alkaline phosphatase immunohistochemistry [10]. Similarly to what we observed for Shh, Netrin-1 signal is highest at the floor plate and decreases rapidly in the first ~50 μm from the floor plate, with a shallow gradient present in the remainder of the spinal cord, which includes the region from the floor plate where Netrin-1 dependent errors occur (relative distance of 0.35–0.6). This gradient shape is reminiscent of the gradient shape for Shh and suggests that the Netrin-1 gradient is also steep close to the floor plate and shallow in the remainder of the spinal cord. However, we were unable to confirm this by more precise quantification using immunofluorescence because the Netrin-1 antibodies that work for immunohistochemistry are no longer available.

Intriguingly, the Shh- and Netrin-1-dependent guidance errors occur in the region of the spinal cord where Shh and most likely Netrin-1 gradients are shallow, not steep (Fig. 1C), indicating that loss of one guidance cue is sufficient to cause guidance defects in shallow gradients. Since guidance defects occur in this shallow gradient region, we hypothesized that having multiple guidance cues may be most important when the fractional change is low, when it is more difficult for a growth cone to obtain an accurate sense of direction from a single gradient.

### *le Massif* Microfluidic Axon Guidance Assay

The guidance of commissural neuron axons towards the floor plate in mice occurs between e9.5 and e11.5 [20,21]. Considering that commissural axons grow at 13–20 μm/h in vivo [21,22] and that the distance from the roof plate to the floor plate is about 500 μm, an individual axon will therefore take ~25–38 h to reach the floor plate. Since neurons vary in when they differentiate and begin their axon outgrowth, we approximate that commissural neurons are exposed to guidance cues en route to the floor plate over 1–2 d.

**Fig 2:**
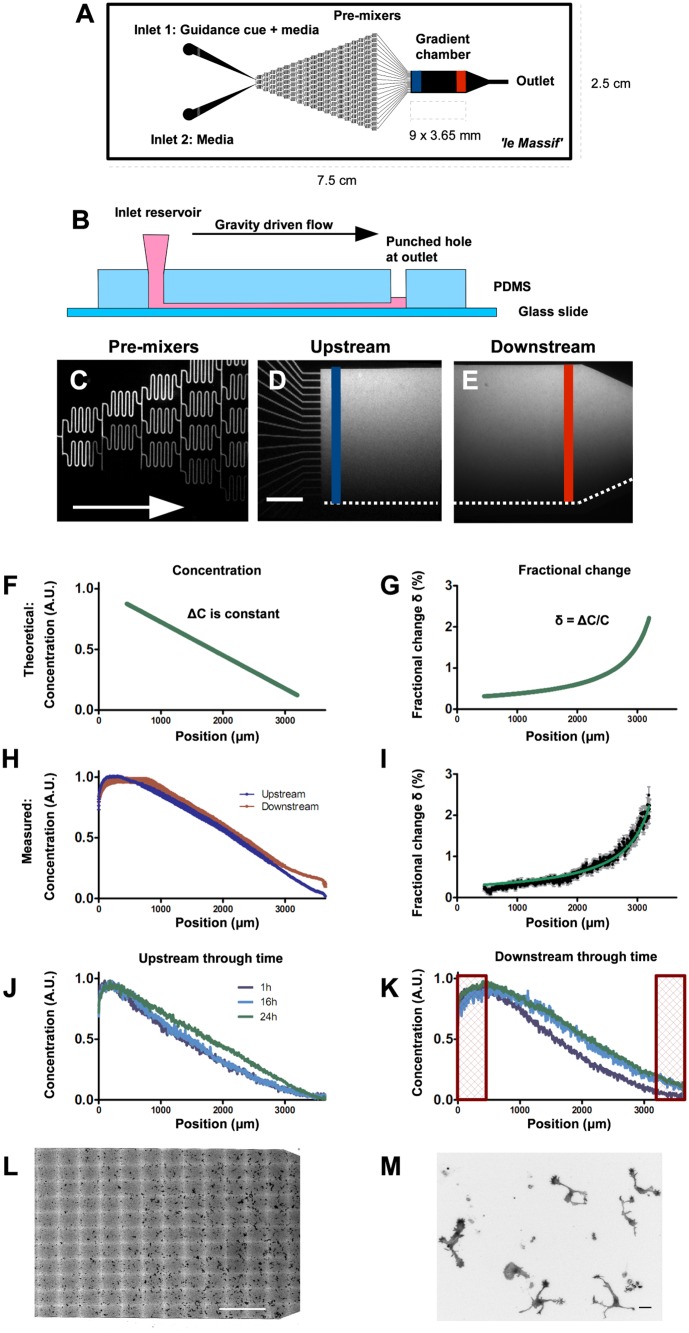
*le Massif* microfluidic gradient generator can produce shallow linear concentration gradients that are stable through space and time. **(A)** Drawing of the *le Massif* microfluidic device. Dimensions of the glass slide and the cell culture chamber are indicated with dashed lines. The blue line represents the upstream limit of the gradient chamber; the red line represents the downstream limit. The device generates linear concentration gradients using a pre-mixing microfluidic paradigm, in which a known concentration of cue is added to a reservoir at inlet 1 and culture media without guidance cue is added to inlet 2. **(B)** Schematic cross-section of *le Massif* microfluidic gradient generator. The fluid-filled inlet reservoirs drive a flow from left to right, with the fluid accumulating in the hole at the outlet. **(C–E)** The gradient visualized using tetramethylrhodamine-conjugated 40 kDa dextran. When the streams from inlets 1 and 2 converge, the concentrations are mixed and divided at 18 discrete steps throughout the premixers **(C)**, generating 20 discrete concentrations that then enter into the gradient chamber **(D)**. The overall gradient shape smoothens rapidly by diffusion to become continuous while maintaining its overall profile until the downstream region (red line in **E**), where the media flows to the outlet. **(F,G)** Theoretical calculation of the concentration **(F)** and fractional change in concentration, δ, **(G)** of guidance cue in the gradient chamber. The fractional change range (0.3 ≤ δ < 2.2%) encompasses the shallow gradients observed in vivo. **(H,I)** Measured fluorescence intensity **(H)** of the upstream (blue line in **D**) and downstream (red line in **E**) limits of the gradient chamber, as well as the fractional change in concentration **(I)** calculated from the upstream concentration profile. Mean ± standard error of the mean (SEM) of 14 independent gradient devices are shown. The measured values match the theoretical predictions (green line in I), with a mean-square-error of 0.091 compared with the theoretical fractional change. **(J,K)** Gradient profile over time for the upstream **(J)** and downstream **(K)** limits. The red boxes in **(K)** represent 450 μm, which are excluded due to the gradient flattening that occurs at the boundaries of the device resulting from the no-slip condition. **(L)** Stitched inverted fluorescence image of the downstream area shown in **(E)**, seeded with commissural neurons that were fluorescently stained with rhodamine-conjugated phalloidin. **(M)** Representative higher-magnification inverted fluorescence image of rhodamine-phalloidin-stained commissural neurons in the gradient chamber. After 45 h in culture, most neurons have extended an axon. Scale bar (**C–E,L**): 1 mm. **(M)**: 25 μm.

We thus developed a guidance assay capable of simulating, over 1–2 d, the shallow Shh gradients that we observed in the spinal cord in vivo. Microfluidic mixing networks allow gradients to be controlled in space and time, allowing for long-term gradients to be established, in contrast to passive source-sink diffusion gradients (e.g., pipette assay and Dunn chamber). We used a linear gradient generator because it allowed us to test a range of fractional change (δ) values. We modified a pre-mixer microfluidic gradient generator [23] by increasing both the length and width of the gradient region, thus maximizing the surface area on which neurons could be exposed to the gradient and thus the sample size. By increasing the width of the gradient, we also decreased the range of gradient steepness to physiologically relevant levels, as determined in vivo (Fig. 1B). Our wider gradient chamber required an increase in the number of sequential mixing channels (Fig. 2A), which offered the added benefit of increasing the overall resistance, thus decreasing the flow velocity and resulting shear stress, which can be harmful to axons [24]. With these device improvements, we were thus able to generate stable, long-term gradients.

In our microfludic device (Fig. 2A), gravity-driven flow (Fig. 2B) directs fluid into the mixing network (Fig. 2C), resulting in a linear gradient throughout the chamber (Fig. 2C–E). We used fluorescent dextran to measure the concentration and fractional change of the gradient, and found that as predicted, the gradient is linear and maintained throughout the chamber (Fig. 2F,H), and stable over a 24 h period (Fig. 2J–K). Furthermore, the measured fractional change (δ) values match the predicted values, ranging between 0.3% and 2.2% (Fig. 2G,I). Since the gradient is linear (Fig. 2F,H), the fractional change increases as the concentration decreases across the device (Fig. 2G,I). The device was biocompatible, as dissociated commissural neurons could be cultured in the device and were observed to extend axons (Fig. 2L,M). To test whether the slow flow rate present in the chamber would bias the direction of axon growth, we measured the angle at which the axon emerged from the cell body and the angle at which the tip of the axon was oriented. We found that the presence of fluid flow did not change the random distribution of these angles (S3 Fig), and therefore the shear stress in our device is negligible and does not bias the direction of axon initiation from the cell body nor the direction of axon growth. Therefore, we have developed an assay, which we named *le Massif*, to challenge commissural neurons with physiologically relevant gradients.

### Axons Turn Up a Gradient of Shh or Netrin-1 in *le Massif*

**Fig 3:**
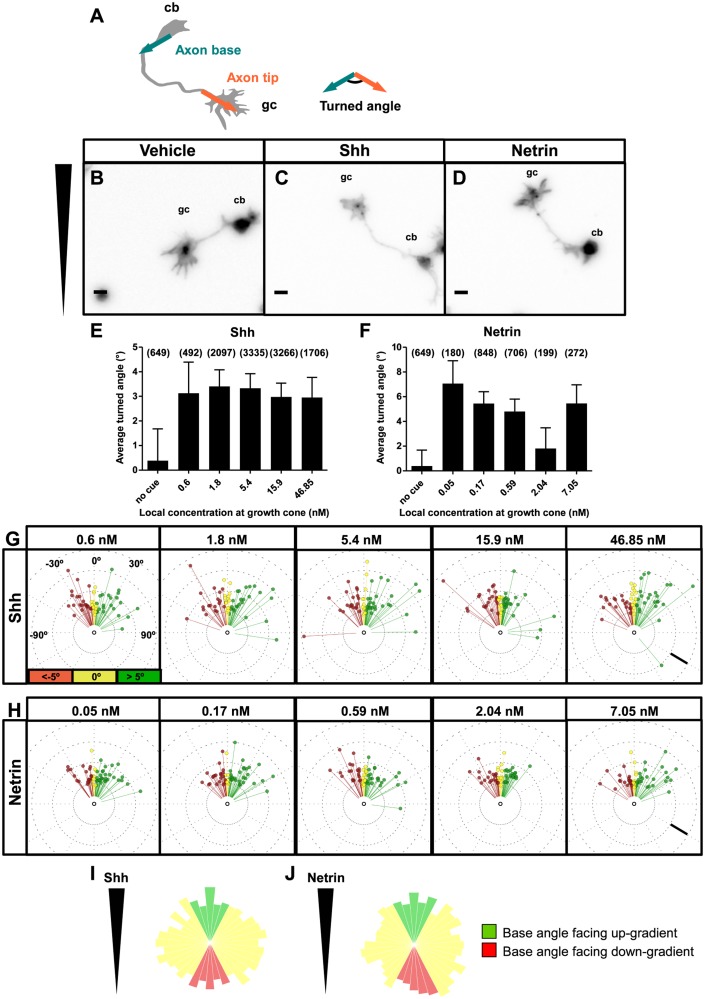
Axons turn up gradients of Shh and Netrin-1 in *le Massif*. **(A)** The turned angle is defined as the angle between the line representing the proximal 20 μm of the axon at the cell body (axon base, turquoise) and the line representing the distal 20 μm of the axon at the growth cone (axon tip, red). The sign of the turned angle is positive if the turn is in the direction of the higher concentration of chemoattractant, and the sign of the turned angle is negative if the turn is in the direction of the lower concentration. **(B,D)** Images of commissural neurons grown for 1 d in culture followed by the application of **(B)** control vehicle gradient (BSA), **(C)** Shh gradient or **(D)** Netrin-1 gradient for 24 h. Wedge represents the gradient orientation. The average turned angles of axons as a function of the absolute concentration of **(E)** Shh and **(F)** Netrin-1 at the growth cone. Axons turn to a similar extent over a wide range of concentrations. The number of axons in each group is indicated in parentheses. **(G,H)** Circular distribution of individual turned angles. The angular deviation from vertical represents the magnitude of the turned angle, such that points to the right of the center are attracted (green) and those to the left are repelled (red). Neurons which turned between -5° and 5° are considered to be neutral (yellow). The distance of each point from the center represents the axon length. A random sample of 60 axons for Shh and Netrin-1 are plotted. A small shift in distribution towards attraction is seen across a wide range of concentrations for either cue. **(I,J)** To exclude the possibility that the gradients influence the angle at which the axon protrudes from the cell body, a gradient was applied 6 h following plating the neurons, before most neurons had initiated an axon. Axon base angle frequency distributions for axons grown in **(I)** Shh and **(J)** Netrin-1 were measured. Green bars represent the number of axons with a base angle facing up-gradient, and red bars indicate axons with angles facing down-gradient. There is no significant bias in angle distribution for either cue (Rayleigh test for uniformity, Shh: *n* = 2,028; Netrin: *n* = 2,805). Scale bars (**B-D**): 10 μm. (**G,H**): 25 μm. Error bars represent SEM. cb, cell body; gc, growth cone. See also S3 Fig.

We established gradients of Shh or Netrin-1 after commissural neurons had been cultured for 24 h, when the majority of neurons had already initiated an axon. We calculated the turned angle of an axon as the difference between the base and tip angles (Fig. 3A) and scored the angle as positive if the axon turned towards the gradient and negative if it turned away. By varying the maximal concentration of ligand in a particular chamber, we could test a wide range of concentrations. In a control gradient (Phosphate buffered saline/BSA), we observed a wide range of turned angles towards and away from the gradient, resulting in a net turned angle of 0° (Fig. 3B,E). Neurons exposed to a gradient of Shh (Fig. 3C,E) or Netrin-1 (Fig. 3D,F), however, turned towards the higher concentration of chemoattractant. For either cue, we observed axon turning in response to a wide range of concentrations at the growth cone (Fig. 3E,F). The distribution of turned angles of individual axons confirmed that wide concentrations of Shh and Netrin-1 induced biases towards attraction (Fig. 3G,H). To eliminate the possibility that Shh or Netrin-1 influences the orientation at which the axon exits the cell body (axon base angle), thus confounding our measurement of the angle turned, we performed experiments where gradients were established 4–6 h after the neurons were plated, before the majority of neurons had initiated an axon. We found that Shh and Netrin-1 gradients induced no significant bias in the distribution of axon base angles facing up-gradient (higher concentration) compared to those facing down-gradient (lower concentration) (Fig. 3I,J). Therefore, *le Massif* generates gradients that can induce axon turning without any effect on axonal initiation.

### Axons Turn More When the Fractional Change in Concentration Is High

**Fig 4:**
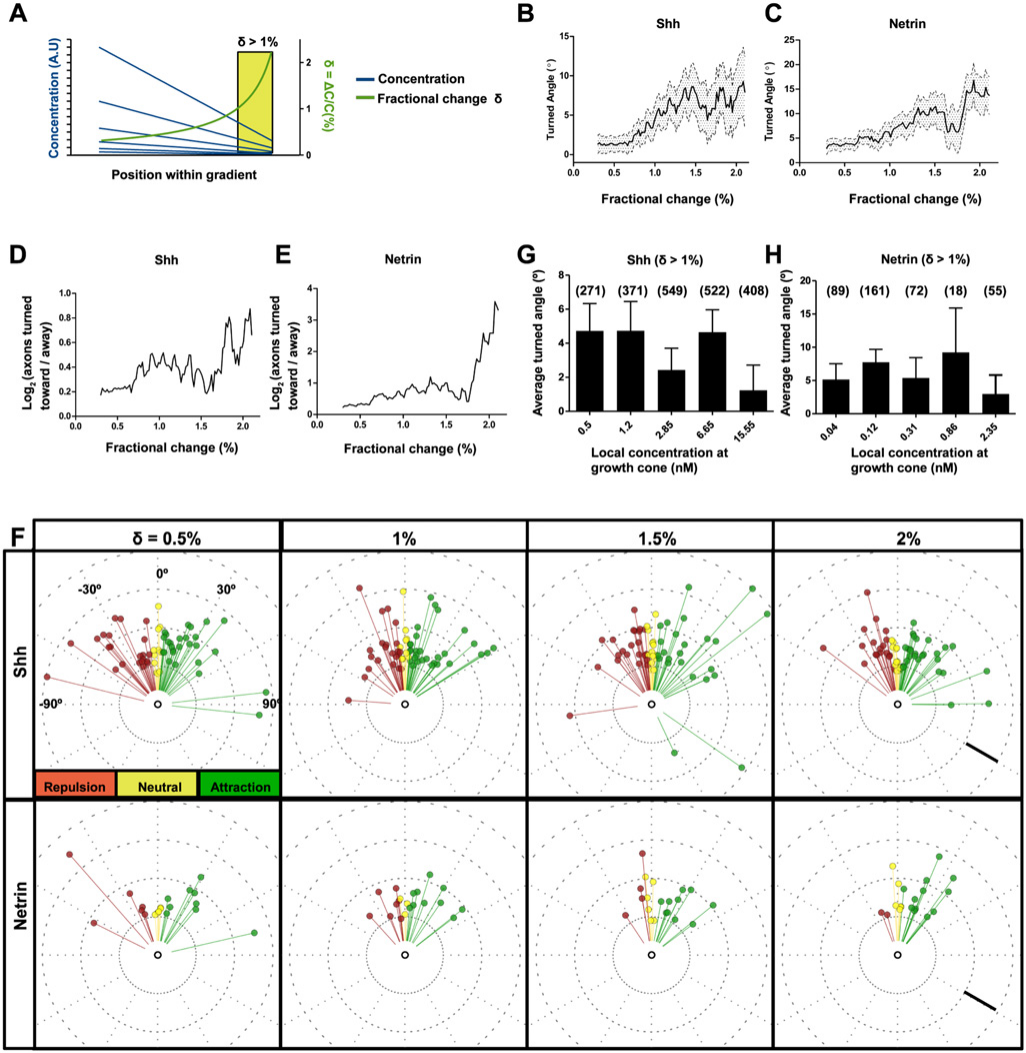
Commissural axon turning depends more on the gradient fractional change than the concentration. **(A)** Schematic of the concentration gradient within the microfluidic chamber. Because the concentration gradient is linear, the fractional change, δ, is highest when the concentration is low. Regardless of the maximum concentration used, the fractional change at a given position in the chamber is the same. **(B–E)** Both **(B)** Shh and **(C)** Netrin-1 gradients induce more turning as the fractional change increases. The average turned angle is plotted as a function of a moving window of 0.5% fractional change. Error bands represent mean and error of all neurons within the window at each position. The slope of the linear regression was 4.5 ± 2.0 degrees/% for Shh and 6.5 ± 2.4 degrees/% for Netrin. This increased turning response corresponds to an increase in the ratio of axons which turned in the correct direction in **(D)** Shh and **(E)** Netrin-1 gradients. (Shh: 43 < *n* ≤ 1,302; Netrin: 17 < *n* ≤ 569 from ≥ 4 independent microfluidic devices). **(F)** Circular distribution of individual turned angles. A random sample of 60 axons for Shh and 20 axons for Netrin-1 are plotted. For either cue, as the fractional change increases, turning is more robust, with a clear bias in the distribution of turned angles towards attraction and a decrease in the proportion of guidance errors. **(G, H)** For fractional change δ > 1%, there is no improvement in turning with increasing absolute local concentration of **(G)** Shh or **(H)** Netrin-1 at the growth cone. The number of axons in each group is indicated in parentheses. One-way ANOVA with Newman Keuls’ multiple comparison test indicated no significant differences between the groups. Scale bar **(F)**: 25 μm. Error bars represent SEM.

Since we observed similar turning over a wide range of concentrations (Fig. 3E,F), we then analyzed axon turning as a function of the fractional change in concentration, δ, across a growth cone. The fractional change is a function of the chamber geometry, and not of the maximum concentration used (Fig. 4A). Therefore, the fractional change is independent of the maximum concentration in the gradient chamber, so long as the minimum concentration is 0. We found that axon turning increased as a function of fractional change for both gradients of Shh and Netrin-1 (Fig. 4B,C). This corresponded with an increase in the ratio of axons that turned towards the gradient compared with those turning away (Fig. 4D,E). Thus, there seem to be fewer guidance errors as the fractional change across the growth cone increases. This was also illustrated with the distribution of turned angles of individual axons (Fig. 4F). At low fractional change, the population of axons have variable turned angles, with a slight bias toward attraction. As the fractional change increased, a bias towards attraction became more pronounced, as there were fewer axons that were erroneously repelled. We then compared the turned angles of axons experiencing the same fractional change (δ > 1%) with different concentrations at the growth cone. When δ > 1%, we observed no trend toward increased turning as the concentration at the growth cone increased (Fig. 4G,H). Therefore, for commissural neurons in gradients of Shh or Netrin-1, the turning response is more sensitive to changes in the fractional change than the local concentration at the growth cone.

### Axons Turn More Robustly in Combined Gradients of Shh and Netrin-1

**Fig 5:**
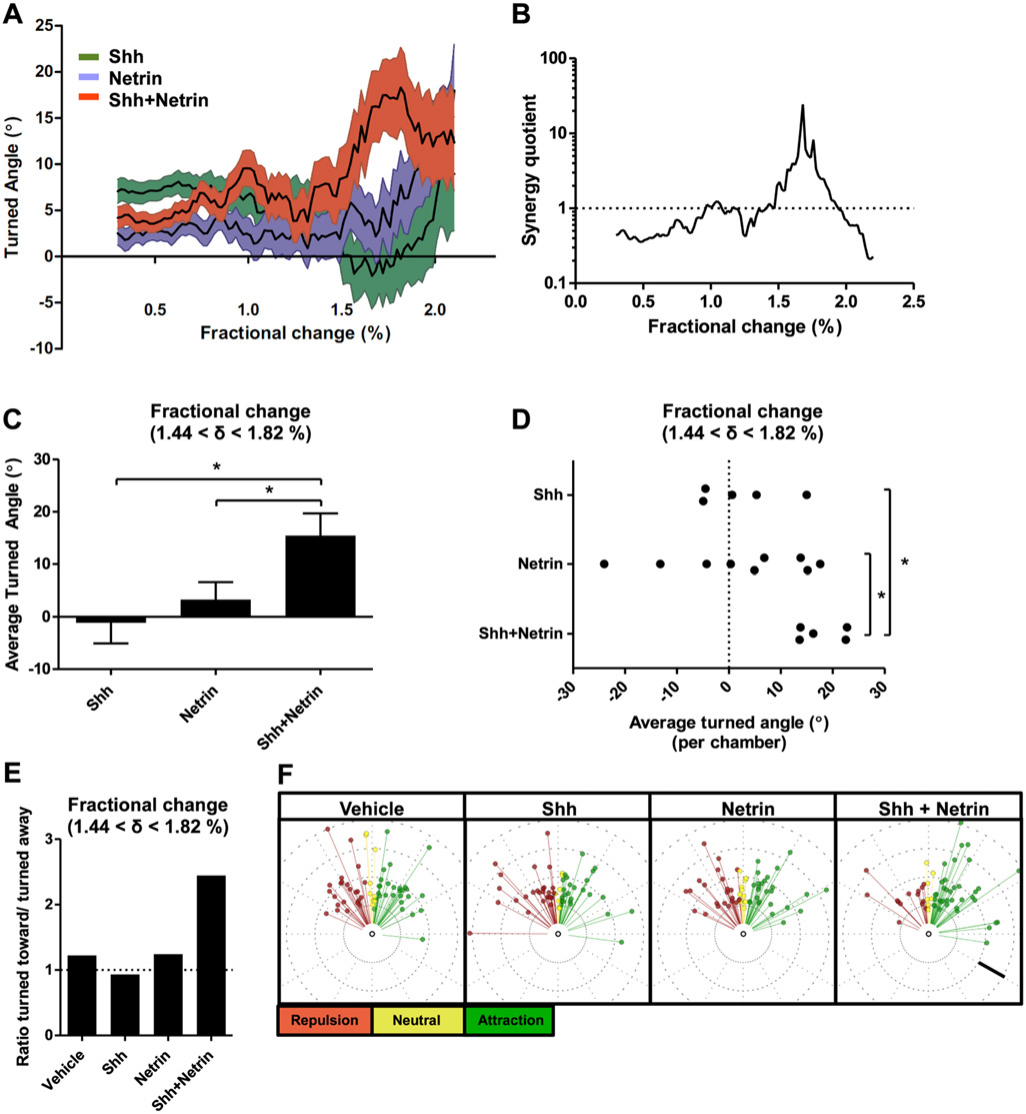
A combined gradient of Shh and Netrin-1 enhances axon guidance when the fractional change is sub-optimal for guidance towards a single cue. **(A)** Average turned angle as a function of fractional change when axons are exposed to gradients for 45 h. Axons exposed to gradients of Shh (green) or Netrin-1 (blue) have a maximum response when the fractional change is at its highest, δ = 2.2%. A combined gradient of Shh and Netrin-1 (red) induce turning to a similar magnitude as either cue individually at δ = 2.2%, but induce turning that is higher than for either cue individually when the fractional change is below its maximum (1.4 < δ < 1.8%). Mean and error of the average turned angle within a window of 0.5% are shown (Shh: 31 < *n* ≤ 1,004; Netrin: 38 < *n* ≤ 1,107; Shh+Netrin: 40 < *n* ≤ 1,164). **(B)** We defined the synergy quotient as the ratio of the turning towards the combined gradient to the sum of the turning to either cue individually (calculated using the means from A). For the majority of the combined gradient, the combined influence is sub-additive. However, the combined cues act synergistically when the fractional change is (1.44 < δ < 1.82%), just below its maximum value. **(C)** At this fractional change (1.44 < δ < 1.82%), axons responded much more robustly than for either cue individually, resulting in a higher average turned angle. **(D)** The synergy observed when 1.44 < δ < 1.82% is consistent between independent experiments (Shh: *n* = 5; Netrin: *n* = 9; Shh+Netrin: *n* = 5; *p* = 0.043 one-way ANOVA with Newman-Keuls Multiple Comparison Test). **(E)** The increase in turned angle results from a larger ratio of axons turning toward than away from the gradient in the combined gradient (Shh: *n* = 55; Netrin: *n* = 92; Shh+Netrin: *n* = 73). **(F)** Angular distribution of the turned angles of a random sample of 60 neurons for 1.44 < δ < 1.82%. No bias in distribution is observed in the control gradient, nor in gradients of Shh or Netrin-1 alone. However, when axons are exposed to the combined gradient, there is a clear bias in the distribution of turned angles towards attraction. Therefore, in the presence of shallow combined gradients, there are fewer guidance errors occurring than for either cue individually. Scale bar **(F)**: 25 μm.

Since guidance errors occur in the region of the spinal cord where Shh and Netrin-1 gradients are shallow, not steep (Fig. 1), we hypothesized that multiple guidance cues might be most important for guiding axons in shallow gradients. Therefore, we next tested whether combining gradients of two guidance cues might modulate the axon turning response in relation to fractional change. We performed guidance assays with 20 nM Shh and 0.69 nM Netrin in the inlet, generating local concentrations ranging from 2.5 to 18.55 nM for Shh and 0.08 to 0.65 nM for Netrin-1, encompassing the range for which we see axon turning (Fig. 3E–H, Fig. 4G,H). Gradients of Shh or Netrin-1 alone and in combination were established 6 h after neurons were plated and maintained for 45 h. In either Shh or Netrin-1 gradients, the turned angle peaked at the highest fractional change, δ = 2.2% (Fig. 5A). Upon applying both Shh and Netrin-1 simultaneously, the average turned angle increased more quickly as a function of fractional change, such that axons were turning robustly in a region of the double gradient where a single cue was not eliciting much turning (1.4 < δ < 1.8%). Interestingly, at the maximal zone of fractional change (1.8 < δ < 2%), there was no observable difference in the angle turned induced by the individual or combined cues (Fig. 5A).

To assess the relationship between the combined cues compared to the individual cues, we calculated the synergy quotient as the turned angle in the combined gradient divided by the sum of the turned angles to both cues individually (described in the Materials and Methods section). With this measurement, a value below 1 indicates sub-additive effects, 1 is defined as additive, while a value above 1 is synergistic. We observed additive and sub-additive effects for the majority of the fractional change range, apart from fractional change range of 1.44 < δ < 1.82%, in which the effect of the combined cues is much greater than the sum of the individual cues, demonstrating synergy (Fig. 5B). Hence the synergistic effect of the combined gradient is greatest when the fractional change is below the maximum.

At this fractional change (1.44 < δ < 1.82%) in which the combined gradients lead to synergy, axons responded much more robustly to the combined cues than for either cue individually, resulting in a higher average turned angle (Fig. 5C). This effect was remarkably consistent, with every independent gradient chamber with combined cues having strong positive turning in this range of fractional change, whereas the gradient chambers with the single cues had variable turned angles with no consistent bias (Fig. 5D). The synergy occurring when the two cues are present was also demonstrated by the larger proportion of axons which turn up the combined gradient than for either cue individually (Fig. 5E). The influence of combining cues on the proportion of correct versus incorrect guidance decisions was also apparent when we observed the distribution of the turned angles in the different conditions (Fig. 5F). For the control gradient (vehicle) and gradients of Shh and Netrin-1, there was no bias towards either attraction or repulsion. Remarkably, when both cues were presented as a combined gradient, there was a clear bias towards attraction, wherein very few axons failed to reorient their direction. Together, these results indicate that a combination of guidance cues can act in synergy to guide axons when the gradient steepness is sub-optimal for the growth cone to sense the direction of a single cue gradient.

### Combined Gradients of Shh and Netrin-1 Induce Polarized Src-Family Kinase Activation in the Growth Cone

**Fig 6:**
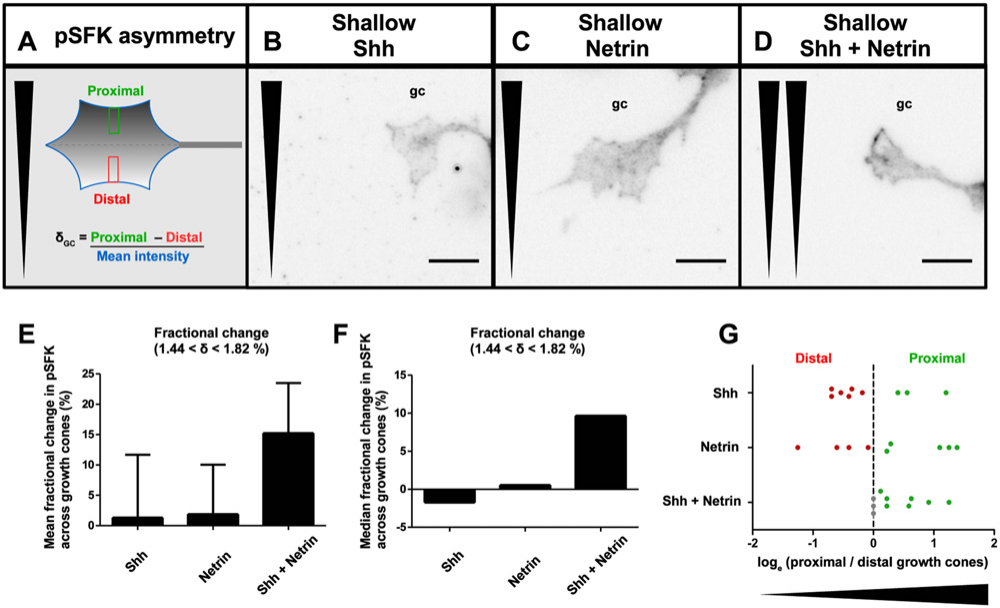
Combined shallow gradients of Shh and Netrin-1 synergize to polarize activated Src family kinase within the growth cone. **(A)** pSFK asymmetry across each growth cone was measured as the fractional change in pSFK fluorescence (δ_GC_). This was defined as the difference between mean intensities in the proximal third (green rectangle) and the distal third (red rectangle) of the mean intensity profile spanning the width of the growth cone, divided by the mean intensity of the entire growth cone area. Growth cones exposed to shallow, 1.44 < δ < 1.82% gradients of **(B)** Shh alone or **(C)** Netrin-1 alone do not show a directional bias in pSFK distribution after 2 h. However, **(D)** in the combined gradient, pSFK is asymmetrically localized to the proximal side of the growth cone (in this example, there is an 18% pSFK fractional change, similar to the mean fractional change that we observe in [**E**]). This results in a higher **(E)** mean and **(F)** median fractional change in pSFK across the growth cones. Polarized pSFK growth cone asymmetry was assessed by using a Wilcoxon signed-rank test against a hypothetical median of 0 (Shh: *n* = 99; Netrin: *n* = 107; Shh+Netrin: *n* = 126; *p* = 0.018 for Shh+Netrin). **(G)** The ratio of the number of growth cones with proximal versus distal pSFK asymmetry for each independent gradient device for 1.44 < δ < 1.82% demonstrates that while the devices with single cue gradients of Shh and Netrin alone have no bias for a proximal versus a distal distribution, those with combined gradients have consistently equal or more growth cones that are proximally rather than distally polarized (*p* = 0.025, Chi-square analysis). Wedge represents direction of gradient (**A–D,G**). Scale bar (**B-D)**: 10 μm. Error bars represent SEM.

Since SFKs act downstream of Shh [15] and Netrin-1 [14] to guide commissural axons, it has been proposed in a recent review by Dudanova and Klein [25] that Shh and Netrin-1 signaling may converge on SFKs. Furthermore, the active form of SFKs, phosphorylated at Y418 (pSFK), accumulates on the side of the growth cone proximal to the higher concentration of Shh and is sufficient to relay the direction of the gradient [15]. Using *le Massif*, we challenged commissural growth cones with gradients of either Shh, Netrin-1, or a combination of both for 2 h, and then assessed the distribution of growth cone pSFK along the direction of the gradient (Fig. 6A). Growth cone pSFK distribution was measured by the fractional change in signal intensity across the width of a growth cone (δ_GC_), which represents the difference in the amount of pSFK at the proximal versus the distal side of the growth cone, relative to the overall levels. Growth cones with more pSFK on the proximal side closer to the higher guidance cue concentration had positive δ_GC_ values, and growth cones with more pSFK on the distal side closer to the lower guidance cue concentration had negative δ_GC_ values. For axons exposed to a fractional change of 1.44 < δ < 1.82% in single cue gradients of Shh (Fig. 6B) or Netrin (Fig. 6C), there was no consistent bias in the direction of pSFK distribution (mean and median δ_GC_ ~0%, Fig. 6E,F). In the combined gradient, however, more growth cones had a proximally biased pSFK distribution (Fig. 6D–F).

This shift towards proximally distributed pSFK was also apparent when we calculated the ratio of the number of proximal to distally polarized growth cones in each independent gradient chamber for 1.44 < δ < 1.82%. Independent chambers with single cue gradients of Shh or Netrin-1 vary between having a net proximal or distal pSFK growth cone distribution. In contrast, for the combined Shh and Netrin-1 gradient, there are consistently more chambers with a net proximal pSFK growth cone distribution and not a single chamber in which there is a net distal pSFK growth cone distribution (Fig. 6G). Therefore, single cue gradients of Shh and Netrin-1 that do not elicit axon turning (Fig. 5), also do not elicit a polarized pSFK distribution (Fig. 6). Remarkably, when the Shh and Netrin-1 gradients synergize to elicit turning, this also corresponds to a higher pSFK distribution on the side of the growth cone facing the high concentration of guidance cues. Taken together with our quantification of gradients and guidance defects in vivo, these results indicate that a combination of guidance cues can act in synergy to polarize growth cones in regions where the gradient steepness is sub-optimal for the growth cone to be polarized by a single cue gradient.

## Discussion

**Fig 7:**
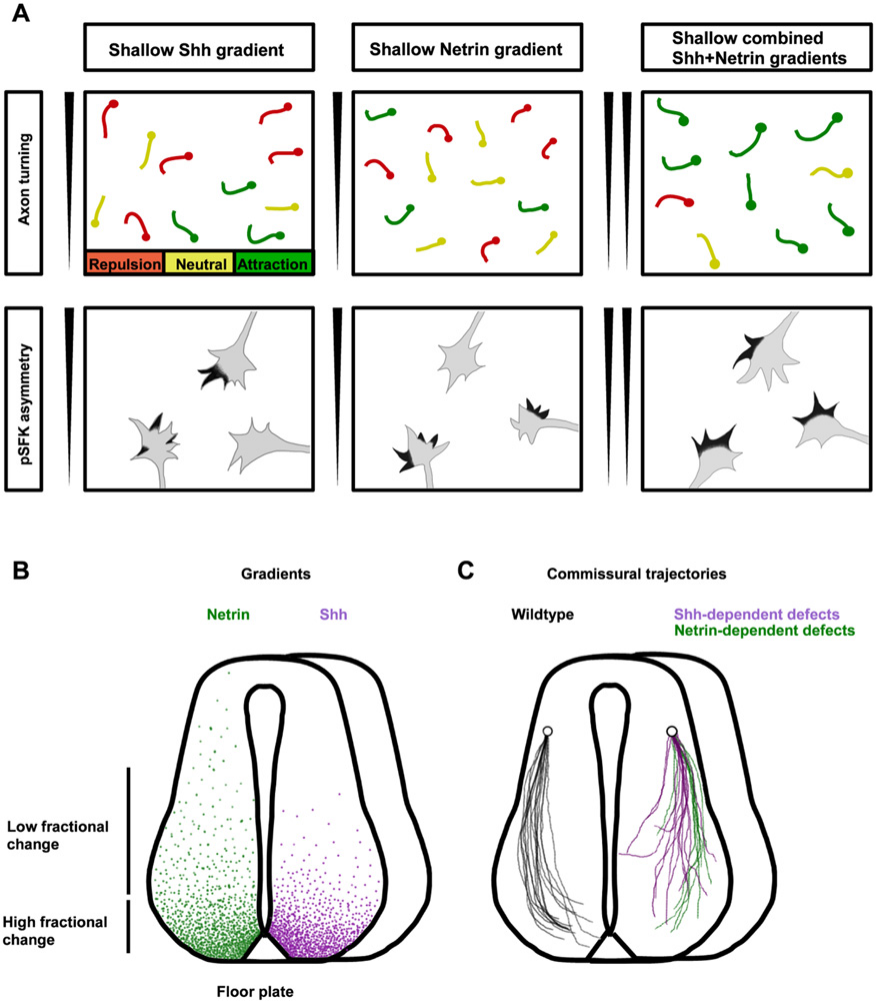
Shh and Netrin-1 synergize to guide commissural axons when their gradients are shallow. **(A)** In shallow individual cue gradients, commissural axon turning is random (left and middle). In the presence of combined Shh and Netrin-1 shallow gradients (right), axons turn toward the higher concentration of guidance cue. When either gradient alone is insufficient to promote axon turning, pSFK activation is also not polarized up the gradient. However, the combined Shh and Netrin-1 gradient biases pSFK distribution to the side of the growth cone facing up the gradient. **(B)** In the developing spinal cord, the gradients of Shh and Netrin-1 proximal to the floor plate are steep (high fractional change), whereas further from the floor plate, the gradients of Shh and Netrin-1 are shallow (low fractional change). **(C)** When either the Shh or Netrin-1 signaling pathway is disabled, guidance errors occur at approximately the midpoint of the spinal cord, where the gradients are shallow. Our results suggest that in this shallow gradient region, integrating multiple guidance cues is necessary for correct growth cone guidance.

In this study, we developed a microfluidic gradient generator that enabled us to directly measure long-term axon turning responses under physiological gradient steepnesses. We demonstrate that commissural axons turn more when the fractional change is high for both Shh and Netrin-1. Additionally, we show that when the steepness of the gradient is limiting, a combined gradient of Shh and Netrin-1 induces axon turning at a gradient steepness at which either cue alone cannot (Fig. 7A). Furthermore, at this same gradient steepness, we observe polarized growth cone activation of SFK only when Shh and Netrin-1 are presented in combined gradients (Fig. 7A). Therefore, we propose that collaboration between Shh and Netrin-1 results in synergy specifically in circumstances in which both gradients are shallow. In this steepness-limited synergy, multiple overlapping signals are necessary for the growth cone to properly interpret the orientation of the gradient when the gradient is shallow. In the developing spinal cord, we propose that this corresponds to a region midway along the commissural axon trajectory (Fig. 7B). Notably, the analysis of the phenotype of four different mouse models (*Netrin-1*, *DCC*, *Boc*, and *Smo* conditional mutants) shows that when one of these pathways is impaired, guidance errors occur in this shallow gradient region (Fig. 7C). Therefore, our data support a model where guidance cue collaboration is essential to guide axons when the gradient steepness is sub-optimal for them to be guided by a single cue.

### *le Massif* Microfluidic Gradient Device Generates Temporally and Spatially Stable, Physiologically Relevant Gradients

An essential component of the current study is the use of microfluidic mixing networks to generate spatially and temporally stable concentration gradients. *le Massif* guidance assay allows us to assess axon turning over the course of days. Since an image only has to be taken at the final time point, *le Massif* is compatible with high-content screening microscopes, allowing assays to be performed in a high-throughput manner, such that a large number of axons can be imaged and analyzed (over 200 per chamber).

An additional advantage of *le Massif* over other axon guidance assays is that it generates gradients with low-to-moderate fractional change, 0.3 < δ < 2.2%, which sits near the lowest fractional change eliciting detectable guidance responses (Fig. 4B,C). This is critical for studying the influence of fractional change on axon turning. This contrasts with techniques such as the pipette assay, which generates gradients with a steep fractional change (5 < δ < 35%) [26]. While printed gradient assays allow precise control over the gradient parameters [19,27–29], the gradient is printed prior to the addition of the neurons, making it difficult to distinguish the effect of the gradient on direct axon turning, rather than differential axon outgrowth or growth rate modulation (a notable exception to this is Mortimer et al. [30], which tested the effect of printing the guidance cue before and after addition of explants). Furthermore, in these assays, axons are either growing along pre-defined corridors or are growing from an explant, making individual axon trajectories often difficult to identify. In contrast, individual axon trajectories can be easily visualized in *le Massif* because the dissociated neurons are grown at low density, so we can clearly measure directed turning of individual axons. Also, by imposing the gradient after axon outgrowth has commenced, we avoid the gradient influencing the orientation of axon protrusion from the cell body [29]. Thus, owing to the versatile process of microfluidic design, we were able to create a customized gradient generator and generate gradients with physiologically relevant steepness that are stable over days.

### Gradient Steepness Is A Critical Determinant of Axon Turning Responses to Guidance Cues

In addition to axon turning, axon growth [28] and growth rate modulation [19,31] are also processes important in guiding axons to their correct targets. Compared to direct axon turning, growth-rate modulation occurs when axons growing up-gradient grow faster than those growing down-gradient [31]. Previous studies have found that gradient steepness affects axon growth [28] and growth rate modulation [19,31]. We found that gradient steepness also influences axon turning, with robust turning observed for steepness δ~1%–2%. This contrasts with what has been reported for growth rate modulation by NGF gradients, where steepness as low as 0.1% is sufficient to bias DRG axon trajectories [19,31]. Consistent with our results, these 0.1% NGF gradients had no effect on direct axon turning [31]. Similarly, growth of axons is also modulated by gradients with steepness of ≥0.4% (1% over 25 μm), possibly also by influencing the growth rate [28]. Therefore, our results suggest that steeper gradients of 1%–2% are required to induce direct axon turning rather than growth-rate modulation, as hypothesized by Mortimer and colleagues [31].

The gradient steepness at which robust turning occurs is ~1%–2%, within a similar range to our estimate of Shh gradient steepness in the spinal cord (Fig. 1B). For Netrin-1, the lack of effective antibodies for Netrin-1 for use in immunofluorescent staining hampers our ability to directly measure the Netrin-1 gradient steepness in the spinal cord. Previously published images of Netrin-1 in the spinal cord are not amenable to precise quantification because they use alkaline phosphatase immunohistochemistry combined with darkfield imaging [10]. However, examination of the pattern of Netrin-1 staining in the spinal cord [10] does show that the Netrin-1 gradient is steeper closer to the floor plate and shallower further away from the floor plate, consistent with what we observe with Shh and consistent with our hypothesis that Netrin-1–dependent guidance errors occur in shallow, not steep, regions of the gradient.

### Additive and Synergistic Cooperation between Guidance Cues

While significant evidence indicates that multiple guidance cues act on the same axons, precisely how these cues converge to regulate the behavior of the growth cone is poorly understood. The response to two combined cues may be additive or synergistic, depending on whether the output is equal to or above the combined response of either cue individually. Dudanova and Klein [25] define additivity as resulting from cues that act in parallel pathways, and synergy as resulting from cues that have crosstalk between pathways.

Additive effects of guidance cues have been observed with ephrin-A and glial cell line-derived neurotrophic factor (GDNF) on lateral motor column (LMC_L_) axons [32], whereas a synergistic attractive response was seen between EphA and GDNF for the same axons [33]. The former demonstrates that ephrin-A and GDNF act in parallel pathways, while the latter demonstrates crosstalk between EphA and GDNF. EphA and GDNF signal through their respective GPI-anchored receptors ephrin-A and GFRα1, and they crosstalk by sharing a common co-receptor, Ret. The co-activation of ephrin-A and GFRα1 through sharing Ret acts as a coincidence detector and generates synergy [33]. The interaction between EphA and GDNF is an example of concentration-limited synergy, as the combination of low concentrations of guidance cues induced turning when neither cue alone was sufficient.

One of our major findings is that synergy occurs between Netrin-1 and Shh to guide commissural axons. In contrast to Bonanomi and colleagues [33], we find that this synergy is steepness-limited rather than concentration-limited. Steepness-limited synergy occurs when the gradient of individual cues is too shallow to guide axons, but a combined gradient of two cues elicits axon turning. We know that in our case the concentration of the individual cues is not limiting because we observe axon turning when the fractional change is high, despite this corresponding to a lower absolute concentration (Fig. 4A). Furthermore, the range of concentrations used in our experiments all elicit axon turning when the steepness is not limiting (Fig. 4G,H). Thus, we demonstrate for the first time that synergy can also be steepness-limited, when the amount of ligand is not limiting but instead the steepness of the gradient is insufficient for the growth cone to estimate the direction of a single cue gradient.

We also identify SFK as a downstream signaling molecule that integrates Shh and Netrin-1 signaling when the two cues synergize. Both Shh and Netrin-1 can activate SFKs, and SFKs are required for Shh and Netrin-1–mediated axon guidance [14,15]. Furthermore, pSFK polarization at the growth cone reflects the direction of the external gradient [15]. Gradients of Shh and Netrin-1 too shallow to guide axons were also insufficient to correctly polarize pSFKs at the growth cone. Only in the presence of Shh and Netrin-1 together was the direction of the gradient correctly reflected by the growth cone pSFK polarization. Hence, activated SFKs appear to be a node where information from the Shh and Netrin-1 gradients are integrated. In addition to synergy resulting from sharing a common co-receptor as for EphA and GDNF, we find that for Shh and Netrin-1, synergy can arise from shared intracellular signaling molecules. Therefore, diverse mechanisms exist through which synergy between two guidance cues can occur, and more mechanisms likely remain to be discovered.

In the developing nervous system, it is likely that many types of synergistic interactions play a role in the correct guidance of axons to their targets. In the developing limb, where guidance cues act at a choice point for motor axons, concentration-limited synergy may be more important than steepness because the gradient is very abrupt. For commissural axons, which climb a shallow gradient of guidance cues over a long distance, steepness-limited synergy may initially be more critical. Later in their journey, when they reach the steep part of the gradient, it appears that one cue alone may be sufficient to guide axons—for example, the axons in *Boc* mutant mice that cannot respond to Shh but by chance make it close to the floor plate do eventually reach the floor plate [8], possibly because of the effect of the steep Netrin-1 gradient in the ventral spinal cord. Thus, it appears that single steep gradients can guide axons over short distances and allow for more precise guidance near the floor plate, whereas midway along the commissural axon trajectory, synergy between shallow gradients of Shh and Netrin-1 allows these gradients to guide axons that are far from the floor plate, thus extending the distance that guidance cues can act in the spinal cord.

## Materials and Methods

### Immunostaining

All animal work was performed in accordance with the Canadian Council on Animal Care Guidelines. Wild type C57Bl6 or *Shh* ^*-/-*^ mouse embryos were sacrificed at e9.5 or e10.5 and fixed in 4% paraformaldehyde (PFA) in phosphate buffered saline for 1–1.5 h at 4°C and cryoprotected in 30% sucrose. 12–20 μm thick serial sections were cut with a cryostat. Sections were rinsed several times in buffered saline, and then treated for 1 h with a blocking solution containing 0.1% Triton X-100 and 10% heat-inactivated goat serum (HiGS). Spinal cord sections were stained with anti-Shh antibody (kindly provided by S. Scales, Genentech) to detect Shh protein [18]. This antibody is specific, as no signal is detected in *Shh* mutant embryos (S1A Fig). The primary antibody was then replaced with a buffered solution containing 1% HiGS and Alexa Fluor 546-coupled secondary antibody (Molecular Probes; 1:1,000) or Cy3 conjugated secondary antibody (Jackson Immunoresearch, 1:1,000) for 1 h. After staining, slides were mounted with Mowiol (Sigma) and allowed to dry for at least 24 h before imaging. Dot blot immunochemistry was performed by pipetting serial dilutions of recombinant human NShh C24II (R&D) onto a glass microscopy slide, followed by the same procedure and reagent concentrations as above.

For pSFK asymmetry assays, guidance cues were added, then the microfluidic devices were returned to the incubator for 2 h, after which they were fixed with 4% PFA at room temperature for 15 min. Phosphorylated Src-family kinase was detected using a phosphospecific (pY418) primary antibody (Invitrogen, 1:1,000), followed by Alexa Fluor 488-coupled secondary antibody (Molecular Probes; 1:1,000). Chambers were imaged with an IXM high-content screening microscope (Molecular Devices) using a 40X Nikon objective.

### Imaging and Analysis of Gradients

Spinal cord cross-sections were imaged on a Leica upright microscope with 10X and 20X objectives at multiple exposure times to ensure that the images contained the entire dynamic range of the gradients that had been revealed by immunohistochemistry. Images were then analyzed with a custom ImageJ macro, which measured the intensity profile along the dorso-ventral axis at five discrete angles ranging from 95° to 105°, emanating from a region just outside the floor plate. This was performed for both sides of the spinal cord for each image (Fig. 1A). The data was then pooled and visualized using a custom MATLAB script to calculate the mean intensity of the Shh gradient. The background fluorescent signal contribution from both the primary and secondary antibodies was determined by measuring the staining intensity in the neural tube of *Shh* ^*-/-*^ littermates, which were processed simultaneously and imaged identically. The background signal was then subtracted from each quantified Shh gradient profile before the fractional change was calculated. To calculate the fractional change of the measured gradient, the mean intensity profile of the regions of interest were fit to a straight line using Open Office Calc (Maryland), and the fractional change calculated from the fit line.

### Microfluidic Device Fabrication

A microfluidic gradient generator [23] was modified to increase the surface area over which the gradient can be applied. Positive relief master molds were fabricated from a 17.78 cm (7 in) chrome photomask (FineLine Imaging, Colorado) by the McGill Nanotools Microfabrication Facility by spin coating SU-8 2050 (Microchem) to a height of 50 μm onto a 15.24 cm (6 in) silicon wafer. The silicon master wafer with positive relief features was exposed to CHF_3_ plasma for 1 min, then treated with 3,3,3 trifluoroperfluoro-octylsilane in a vacuum desiccator for 30 min to ensure that the polydimethylsiloxane (Silgard 184—PDMS) would not stick to the SU-8 features. PDMS was then mixed thoroughly as per manufacturer's recommendations (10:1 base polymer: curing agent) before being degassed for >15 min in a vacuum and poured onto the silicon master wafer. The PDMS was cured for >3 d at 60°C. The PDMS was then peeled off from the master and cut to individual chips. Through-holes at the two inlets and outlet were made using a biopsy punch. Glass slides (Schott Glass D) were soaked in concentrated nitric acid for 24–36 h, before being rinsed in milliQ water 12 times over 2 h and sterilized by baking at 225°C for 4–6 h. On the day prior to beginning the experiment, both glass slides and PDMS chips were exposed to an oxygen plasma (Plasmaline 415 Plasma Asher, Tegal Corporation, 0.2 mbar for 30 s at 75 W) before bringing the surfaces into contact to form an irreversible bond. Within 20 min following bonding, devices were filled with 0.1 μg/ml poly-D-lysine (PDL; Sigma) to generate an adhesive substrate onto which neurons could attach. After coating for 1 h, the PDL was removed and the microfluidic chamber rinsed twice by adding sterile milliQ water to the outlet. Fluid reservoirs were crafted by cutting the bottoms from 200 μl PCR tubes and positioning the tubes into the punched holes such that both tubes were an equal height. The tubes were both filled with 200 μl of Neurobasal media containing serum, generating a forward gravity-driven flow, which was left to further rinse the PDL coated channels overnight.

### Premixer Microfluidic Gradient Generator

The range of ligand concentration imposed on the axons in the gradient chamber depended on the guidance cue concentration added to the reservoir at inlet 1 (Fig. 2A). The reservoir at inlet 2 was filled with culture media without guidance cue. To visualize and quantify the gradient, we used 40 kDa tetramethylrhodamine-dextran. Hydrostatic pressure was created by filling the inlet reservoirs higher than the outlets (Fig. 2B), which drove fluid flow uni-directionally from left to right throughout the device. When fluid from the two inlets converge, the concentrations at inlet 1 and inlet 2 are mixed and subsequently divided to three discrete concentrations (Fig. 2C). This mixing and splitting occurs a total of 18 times, generating 20 discrete concentrations that are spaced at linear gradations between the concentration at inlet 1 and inlet 2 (no cue). The 20 discrete concentrations then flow from the premixer channels into the gradient chamber, where they meet and diffuse to establish a linear concentration gradient (Fig. 2C). Because the fluid volume is on the microliter scale and the Reynold's number is low (Re < 1), the flow is laminar and there is no convective mixing [34]. Because diffusion is slow over long distances, the diffusion of the guidance cue is slow relative to the flow velocity and the gradient remains linear for the entire 9 mm length of the gradient chamber (Fig. 2E) as long as there is a continuous flow driving the mixing. Consequently, long-term gradients can be maintained without actively controlling the flow rate, so long as the reservoir at the outlet is emptied periodically (approximately every 24 h). The upstream and downstream regions of the gradient chamber were imaged using a 2.5X objective on an upright fluorescence microscope (Leica).

### Primary Commissural Neuron Culture

Commissural neurons were prepared from the dorsal fifth of E13 rat neural tubes as described previously [15,35]. Cells were re-suspended in plating media composed of Neurobasal (Gibco) supplemented with 10% heat-inactivated FBS and 2 mM GlutaMAX (Life Tech). 50 μl of plating media was added to both inlet reservoirs and 50 μl of cell suspension (3,160,000–5,630,000 cells/ml) was added to the outlet. One of the inlet reservoirs was removed and a reverse flow induced by connecting a syringe to the inlet hole via a short rubber hose and pulling on the plunger. While observing with an inverted microscope, neurons were drawn into the gradient chamber, after which the flow was stopped by releasing the plunger, disconnecting the syringe, and then returning the reservoir to the hole. After 4–6 h, inlet reservoirs were filled with 200 μl of plating media, again inducing a forward flow. Approximately 15 h later, the plating media was replaced with serum-free growth media composed of Neurobasal (Gibco) supplemented with 2% B27 (Gibco), 2mM GlutaMAX (Gibco) and penicillin/streptomycin (Gibco).

### Guidance Assay

Shh guidance experiments were performed using the recombinant human NShh C24II (R&D). Netrin-1 guidance experiments were performed using the VI,V peptide [36], which was a kind gift from Dr. Tim Kennedy. The guidance assay was started within 24 h of plating, when most of the neurons had initiated a neurite. Culture media (200 μl) was added to one of the inlet reservoirs and guidance cue or vehicle control (0.1% BSA; Sigma) diluted in culture media (200 μl) to the other. Gradient devices were then returned to the incubator until the following morning (~20 h following gradient application), at which point a Pasteur pipette was used to remove any fluid which had accumulated in the outlet. The devices were then returned to the incubator for a further 4 h, for the remainder of the 24 h assay. Guidance assays over 45 h were performed as described above, except the gradient was established 4–6 h after the neurons were loaded into the device.

The assay was ended by quickly removing all culture media from the inlets and outlets by aspiration and adding 4% PFA (100 μl) to the outlet reservoir. After 15 min, the PFA was removed and replaced with a staining buffer consisting of DAPI (Sigma, 1:10,000) to stain cell nuclei, TRITC-phalloidin (Molecular Probes, 1:250) to stain F-actin, and Triton (Sigma, 1:400) to permeabilize the cells. Neurons were left to stain overnight (~12 h) and the staining buffer was replaced with buffered saline for 1–2 h before imaging.

### Image Acquisition and Analysis of Axon Turning

Fixed specimens were imaged using an IXM high content screening automated microscope (Molecular Devices) with a laser-based auto-focus and a 20X objective (Nikon). To include the entire area of the gradient chamber, 275–300 images were obtained for each device using MetaExpress imaging software (Molecular Devices). All analyses were performed by an observer naive to the gradient conditions for each device. For each image, we traced all isolated axons in each field of view using a custom ImageJ macro. So that the observer would be blind to the direction of the gradient, every image had a 50% chance of being flipped vertically when opened. Image files were analyzed by Flatworld Solutions (Bangalore).

All calculations of neuron position, concentration, fractional change, axon length, and turned angle were performed using a custom MATLAB script. We defined the axon base angle as the angle between the proximal 20 μm of the axon and the direction of flow, and the axon tip angle as the angle between the distal 20 μm of the axon and the direction of flow (parallel to the arrow in Fig. 2C). We defined the turned angle as the difference between the base and tip angles of the axon, where the sign of the difference was positive if the axon turned toward the gradient and negative if the axon turned away from the gradient (Fig. 3A). We considered only axons that faced against the direction of the flow, and we excluded those facing directly towards or against the gradient (within 20° of the gradient direction). We excluded from further analysis axons that were shorter than 20 μm. Because of the local flattening of the gradient near the boundaries caused by the no-slip condition, we excluded any neurons positioned within 450 μm of either boundary (red boxes Fig. 2K).

To estimate the concentration of guidance cue at each growth cone position, we calculated each neuron’s position relative to the gradient chamber, and thus relative to the gradient itself. We assumed a growth cone width of 10 μm for fractional change calculations, which we calculated using the difference in concentration between a point 5 μm above and 5 μm below the neuron, divided by the concentration at the neuron's position. All included growth cones experience fractional change within the range 0.3 ≤ δ < 2.2%.

To calculate the synergy quotient, we first calculated a central moving average (CMA) of the turned angle of all axons within a window of 0.5% fractional change for each Shh, Netrin, and the combined gradient Shh+Netrin (Fig. 5A). We then calculated the synergy quotient (SQ) as:

$${\text{SQ = CMA}}_{\text{Shh+Netrin}}\text{/}\left({\text{CMA}}_{\text{Shh}}{\text{+ CMA}}_{\text{Netrin}}\right)\text{.}$$

### Measurement of Growth Cone pSFK Asymmetry

After being exposed to the gradient(s) for 2 h, neurons were fixed and stained for pSFK. Chambers were imaged with an IXM high-content screening microscope (Molecular Devices) using a 40X Nikon objective. Each growth cone was outlined. Then a line was placed spanning the width of the growth cone outline, parallel to the direction of the concentration gradient. The intensity profile was measured across five parallel lines spaced 1 pixel apart. The average intensity profile of the five lines was then processed using a custom MATLAB script. The fractional change in staining intensity across each growth cone (δ_GC_) was then calculated as the difference in mean intensity between the proximal and distal thirds, divided by the mean intensity of the entire area of the growth cone (Fig. 6A). This value was scored as positive if the higher staining intensity was on the side of the growth cone proximal to the gradient and negative if the higher staining intensity was on the side of the growth cone distal to the gradient.

### Statistical Analysis

All analysis of variance, Chi-square and Wilcoxon signed-rank tests were performed using Graphpad Prism 5 (La Jolla, CA). All Rayleigh tests for unimodal deviation from uniformity were performed using the circStat toolbox for MATLAB.

### Data Visualization

The majority of graphs were generated using GraphPad Prism or Open Office Calc (The Apache Software Foundation), unless otherwise mentioned. Tricolor radial scatter plots (Figs. 3G,H, 4F, 5F) and radial frequency histograms (Figs. 3I,J, S3) were scripted manually with Processing, an open-source sketchpad software (www.processing.org). Random samples of axons were generated with a Processing script using a uniform probability distribution, wherein each axon was equally likely to be selected as the next data point was plotted, and the same data point could not be plotted twice.

## Supporting Information

S1 Data Individual data points underlying the graphs of Figs. 1B, 2F–K, 3E–H, 4A–F, 5A–F, 6E–G, S1, S2, S3, and S1 Table. (XLSX)

S1 Fig Related to Fig. 1. The Shh gradient profile is similar between embryos and does not depend on the concentration of the primary antibody. (A) The anti-Shh antibody specifically recognizes Shh. The Shh staining present in WT floor plate and spinal cord was not present in *Shh* ^-/-^ e10.5 embryos. (B) Quantification of the Shh gradient on a relative distance scale, where the mean gradient profiles from three different e10.5 embryos are shown. The mean value is indicated by the thick black line. (C) Quantification of the Shh gradient profile at antibody concentrations of 1:1,000 (*n* = 3) or 1:2,000 (*n* = 8). The intensity profiles are almost entirely overlapping, despite a 2-fold change in primary antibody concentration. Error bands represent SEM. (D) Quantification of the relationship between Shh concentration and fluorescence intensity by dot blot. Error bars represent SD (*n* = 5). Scale bar (A): 100 μm.
(EPS)

S2 Fig Related to Fig. 1. The measured gradient profile does not depend on the exact position of measurement. (A) We compared intensity profiles measured using wedges in the spinal cord of e10.5 mouse embryos (as in Fig. 1A) with measurements made more laterally (overlapping with Tag-1 positive axons) on the same images from the same sections. (B) Quantification of Shh protein intensity as a function of the relative distance from the floor plate to the roof plate. Although the measurements made laterally appear noisier, the two gradient profiles look similar.
(EPS)

S3 Fig Related to Fig. 3. The flow does not bias the direction at which the axon originates from the soma, nor the axon tip orientation. The base angle and tip angle were measured as described in Fig. 3A. Circular frequency distribution for the axon base and tip angles in gradient devices with no flow or a control (BSA) gradient for 24 h or 48 h are shown. There is no significant directional bias for any of the distributions (Rayleigh test for uniformity, *n* ≥ 403).
(EPS)

S1 Table Related to Fig. 1. Relative position of Shh and Netrin-1 signaling dependent guidance errors. Using previously published images from mice genetically deficient for Netrin-1 (*Netrin-1* ^*-/-*^ and *DCC* ^*-/-*^) or Shh (*Wnt1-Cre;Smo* ^*null/conditional*^ and *Boc* ^*-/-*^) signaling, we determined at what relative position along the spinal cord guidance defects occur for commissural axons by measuring the relative distance from the floor plate to the roof plate at which misguided axons begin to deviate from their normal trajectory. We used these relative distances to define the range of guidance errors.
(XLSX)

## Abbreviations

δ: gradient steepness = fractional change in concentration
δGC: fractional change in signal intensity across the width of a growth cone
ΔC: absolute change in concentration
BMP: bone morphogenic protein
BSA: Bovine Serum Albumin
C: concentration
cb: cell body
CMA: central moving average
e10.5: embryonic day 10.5
fp: floor plate
GDNF: glial cell line-derived neurotrophic factor
gc: growth cone
nc: notocord
PFA: Paraformaldehyde
pSFK: phosphorylated Src-Family Kinase
SFK: Src-Family Kinase
Shh: Sonic Hedgehog
VEGF: vascular endothelial growth factor
